# Extranodal NK/T-cell lymphoma, nasal type (angiocentric T-cell lymphoma): A review about the terminology

**DOI:** 10.4103/0973-029X.80016

**Published:** 2011

**Authors:** Rashmi S Metgud, Jitendra J Doshi, Sarang Gaurkhede, Ravindra Dongre, Ravindra Karle

**Affiliations:** *Department of Oral-Pathology, Rural Dental College, Pravara Rural university, LONI, Tal-Rahata, Ahmednagar, Maharashtra, India*; 1 *Department of E.N.T, Rural Dental College, Pravara Rural university, LONI, Tal-Rahata, Ahmednagar, Maharashtra, India*; 2 *Department of General-Pathology, Rural Dental College, Pravara Rural university, LONI, Tal-Rahata, Ahmednagar, Maharashtra, India*

**Keywords:** Aggressive, angiocentric, angiodestructive, atypical cells, malignant clinical course, midline lethal granuloma, necrosis, NK/T-cells

## Abstract

Extranodal NK/T-cell lymphoma, nasal type (ENKL) is a rare lymphoid neoplasm, which in the past has been grouped with a variety of granulomatous diseases. It is an aggressive non-Hodgkin’s type characterized clinically by aggressive, nonrelenting destruction of the midline structures of the palate and nasal fossa. Despite the malignant clinical course, histological diagnosis can be difficult because of extensive tissue necrosis and multiple biopsies that are often required and has an ominous prognosis, as the average survival rate is between 6 and 25 months as reported with a large number of Asian studies. Several American and European studies have shown similar results. This is the case report of a 60-year-old male patient who presented with nasal obstruction and foul smelling, ulcerative lesion over the palate of 6 months duration, which had been treated with antibiotics and anti-inflammatories without success. After performing a number of diagnostic tests, it was found histologically and confirmed by immunohistochemical analysis that the patient had an ENKL, nasal type (also known as angiocentric T-cell lymphoma).

## INTRODUCTION

Lymphomas account for 3-5% of all malignant tumors; non-Hodgkin’s lymphomas (NHL) account for 60% of all lymphomas. Involvement of the nasal cavity and paranasal sinuses by these tumors is uncommon.[1]

Extranodal NK / T-cell lymphoma, nasal type (ENKL) are aggressive, locally destructive midfacial necrotizing lesions characterized by extranodal involvement, particularly the nasal/paranasal area and represent about 75% of all nasal lymphomas, the rest being B-cell lymphoma[2,3] The lesion typically causes local destruction of cartilage, bone and soft tissues. Lesions may arise de novo at the site or represent a localized progression through stages.[4]

Compared with nodal lymphomas, sinonasal tract lymphomas are difficult to identify correctly because of the scantiness of the biopsy size and because of the problem that extranodal lymphomas generally present in diagnosis. Additionally, diagnostic confusion may result from the variety of pathologic terms that have been applied to this lesion over the years, including midline lethal granuloma, polymorphic reticulosis, midline malignant reticulosis, idiopathic midline destructive disease and lymphomatoid granulomatosis.[5] The mechanism of tumorgenesis remains unclear for ENKL, and future studies are needed to clarifythe molecular pathology of these diseases. Epstein-Barr virus (EBV) is found in most cases of NK-cell leukemia/lymphoma, suggesting an oncogenic role, but patients may have biclonal or polyclonal populations of malignant cells based on differential EBV genome incorporation[2]

These lymphomas are uncommon neoplasms in the United States, representing approximately 1.5% of all lymphomas. A higher incidence, however, has been reported in Asian and South American countries, especially Peru. In these areas, primary NHL accounts for approximately 6.7-8.0% of all lymphomas.[5]

ENKL is typically observed in adults but may be seen in children. Studies have shown a male to female ratio of 2:1 to 3:1. Tumors are most common in the nasal cavity but other sites may include the skin, GIT, testis, kidney, upper respiratory tract and rarely the eye/orbit. Involvement of the regional lymph nodes is unusual until the tumor disseminates.[3]

The initial signs and symptoms are often localized to the nasal region and include nasal obstruction and chronic rhinorrhea. Nasal septal perforation has been reported in 40% of cases. Pain may accompany the nasal symptoms. Systemic symptoms such as fever, weight loss, night sweats and anemia are not typically noted except in advanced cases.[6] Swelling of the soft palate or posterior hard palate may precede the formation of a deep, necrotic ulceration, which usually occupies a midline position. This ulceration enlarges and destroys the palatal tissues, which typically creates an oronasal fistula.[7] The clinical course of NK-cell lymphoma varies with the clinical stage. Patients with limited stage disease (usually nasal disease) typically have an indolent course with tumor restriction to the original site, but others with advanced stage suffer rapid progression to systemic dissemination often accompanied by hemophagocytosis or disseminated intravascular coagulation.[2]

Histopathological examination of the lesion exhibits cellular picture, which is pleomorphic with many large or immunoblast-like cells and relatively few small lymphocytes. A striking feature is the angiocentric distribution of the tumor cells and angiodestruction, which mimics vasculitis.

The infiltrates tend to invade the vascular walls of normal tissue with resultant thrombosis and widespread necrosis. Complicating the histological picture is anintense inflammatory reaction and frequently a proliferation of histiocytes induced by the neoplastic cells.[2]

## CASE REPORT

A 60-year-old male patient reported with a chief complaint of inability to swallow food since 6 months. He had an history of smoking tobacco”; 4^th^ Line “h/o” as “history of since 20 years. Patient’s previous medical records disclosed h/o of nasal obstruction on and off and anosmia and histopathological diagnosis of previous biopsy was given as round cell tumor by a private lab and as tissue insufficient for reporting by TATA memorial hospital.

On general examination, patient’s health was poor, pallor was positive (++) and his body weight was 35 kgs (ht 155cms). He had trismus and on intraoral examination with tongue depressor, oral hygiene was poor and large defect was seen in the secondary hard and soft palate with massive destruction of the tissues. The palatal bone was denuded of its mucosal covering and was seen exposed on the left side of the cleft like defect. Ulcer was foul smelling and oroantral fistula draining pus was also present. Ryles tube feeding was started to maintain nutrition.

Routine blood and urine investigations were carried out. Erythrocyte sedimentation rate (by Westerngren method) was 20 mm/1 hr (normal 0-9 mm/1 hr), hemoglobin level was 8.7 g/dl (normal 13-17 g/dl), TLC and DLC and platelet count were within normal limits. In peripheral blood smear- RBCs showed mild anisocytosis with hypochromia, microcytes (positive), few macrocytes were seen, WBC series showed neutrophilia, toxic granules were seen in neutrophils, platelets were adequate and parasites were absent. Plasma glucose level and serum sodium, potassium, urea, creatinine were within normal limits. With a presumptive diagnosis of either an infective or neoplastic lesion biopsy was done and histopathologically evaluated. On microscopic examination biopsy specimen showed necrotic tissue only without infective organisms or neoplasia and deeper biopsy was advised.

VDRL/RPR test, HIV- Tridot test were nonreactive and Hbs Ag was negative. AFB/ZN staining for acid-fast bacilli was negative. Ultrasonographic findings of abdomen and pelvis and chest X-ray findings were within normal limit. Skin smears from right and left ear lobes and dorsal aspect of right middle finger showed no evidence of Hansen’s disease.

X-ray PNS showed mucosal thickening in right maxillary sinus and both the frontal sinuses appeared normal. X-ray nasopharynx showed anterior osteophytes in the cervical vertebrae suggestive of cervical spondylosis.

Negative microbiological findings ruled out infectious nature of the lesion. Syphilis was excluded by serology. Normal chest radiography and absence of renal involvement ruled out diagnosis of Wegener’s granulomatosis.

On microscopic examination, hematoxylin and eosin-stained sections from deeper biopsy specimen revealed stratified squamous epithelium, which was hyperplastic.

**Figure 1:**
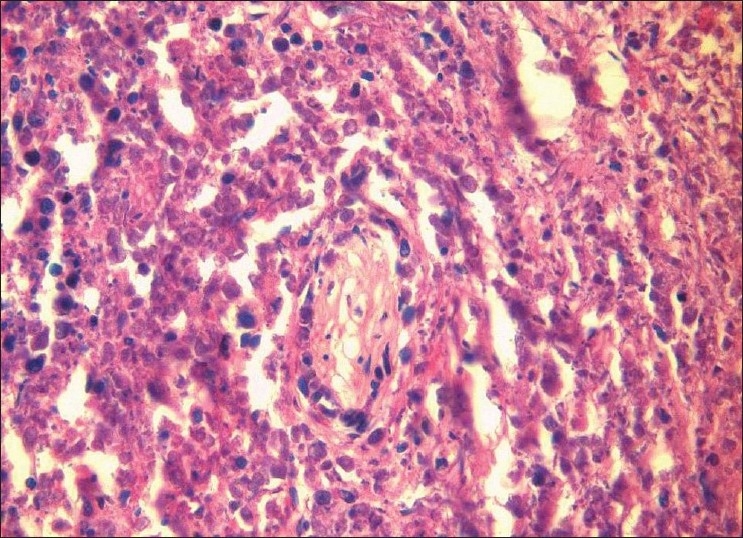
Photomicrograph showing angiocentric (around the blood vessels) distribution of atypical lymphoid cells with hyperchromatic nuclei infiltrating the wall and filling the lumen of a blood vessel (H and E 40×)

**Figure 2:**
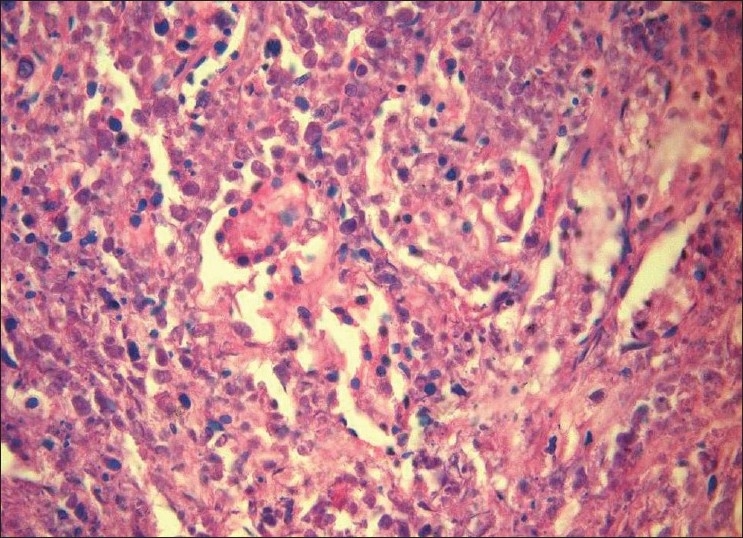
Photomicrograph showing angiocentric (around the blood vessels) distribution of atypical lymphoid cells with hyperchromatic nuclei infiltrating the wall and filling the lumen of a blood vessel (H and E 40×)

**Figure 3:**
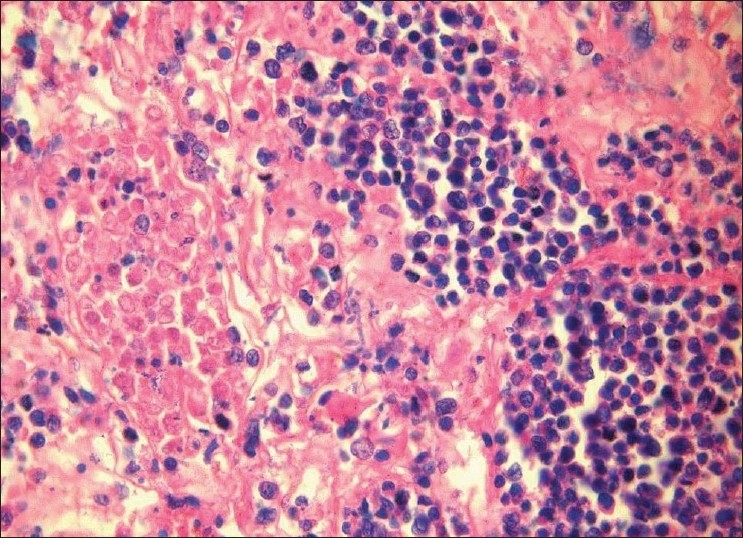
Photomicrograph showing areas of necrosis and atypical lymphoid cells with hyperchromatic nuclei (H and E stain 40×)

Underlying connective tissue stroma revealed lymphoproliferative process characterized by an extremely polymorphous picture; it largely consisted of a mixture of atypical large and small lymphoid cells, along with plasma cells, histiocytes and occasional eosinophils. The large lymphoid cell component was predominant. The large lymphoid cells had hyperchromatic nuclei. The neoplastic cells showed a tendency to be angiocentric with focal areas of vascular destruction [Figures 1 and 2]. Invasion of vascular walls and occlusion of lumina by lymphoid cells (angiodestructive) with varying degrees of cytologic atypia were also noted. The vascular occlusion was associated with prominent necrosis of both tumor cells and normal tissue [Figure 3]. No granulomatous lesions or giant cells were found.

Histological features of invasion of vascular walls of normal tissue by the neoplastic cells and widespread necrosis ruled out diagnosis of midline idiopathic destructive disease. Sections of tissue specimen were subjected for immunohistochemical evaluation. On immunohistochemical analysis tumor cells were strongly positive for leukocyte common antigen (LCA) and negative for CD20 and CD30 and CD3 was equivocal.

Based on clinical findings of localized destruction of the soft and hard tissues confined to the nose and paranasal sinuses, histological features of angiocentric distribution, angiodestructive nature of neoplastic cells and associated necrosis of the normal tissue and the tumor cells, the presence of immunoreactivity with markers indicative of a neoplasm of lymphoreticular origin (LCA, CD3) and absence of renal and pulmonary involvement the diagnosis of “Extranodal NHL -Nasal type” (angiocentricity) was established.

Patient was referred to the Oncology department for management. Bilateral superior deep cervical lymph nodes were palpable. Clinical staging of the lesion was done as T_4_ N_1-2_ M_0_. Since the destructive process was widespread; chemotherapy was planned for the patient. Treatment of the lesion was planned with six cycles CHOP (Cyclophosphomide, doxorubicin, vincristine and prednisone) chemotherapy.

As the patient was poor, treatment was started with Cyclophosphamide inj 500 mg i.v. slow, Vincristine inj 2 mg i.v. slow, weekly for 4 weeks, tab prednisone 120 mgday 1 to day 5(D1-D5). Whole cycle was to be repeated after 28 days. If general condition of the patient improves addition of inj. doxorubicin was planned. But after the first cycle of chemotherapy patient died of clinical complications.

## DISCUSSION

Malignant lymphomas of the head and neck region that originate in the nasal cavity, paranasal sinuses and hard palate form an interesting and frequently diagnostically difficult group.[7] In the past, these lymphomas have been confused with a number of infectious, autoimmune, or inflammatory designations, most of which we now know represent peripheral T-cell lymphomas or angiocentric immunoproliferative lesions. These older terms include midline lethal granuloma, polymorphic reticulosis and midline malignant reticulosis.[5]

**Table:** Different descriptive titles applied to proliferative, ulcerative and midline lesions

1897 McBride	Ulceronecrotic proliferative lesions of the upper airways
1933 Stewart	Lethal granulomatous ulceration of the nose
1939 Wegener	Necrotizing granulomatous process of the mid face
1966 Eichel *et al*	Polymorphic reticulosis
1967 Ah Moo	Midline granuloma
1969 Weissfeld and Shosheim	Lethal midline granuloma
1969 Kassel *et al*	Midline malignant reticulosis
1978 Friedmann *et al*	Lethal midline granuloma syndrome
1972 Leibow *et al*	Lymphomatoid granulomatosis
1984 Jaffe	Angiocentric immunoproliferative lesion
1992 Maxymiw *et al*	Lymphoma presenting as a midfacial necrotizing agent
1992 Grange *et al*	Centrofacial malignant granuloma
1994 Mishima *et al*, Weiss *et al*	Nasal T-cell lymphoma.[8]

Lethal midline granuloma was a disease of undetermined neoplastic significance occurring in the midfacial area and biopsy specimens from these lesions exhibited marked necrosis with inflammatory changes.[2]

The consensus among most investigators is that the term midline lethal granuloma should be used only as a descriptive designation of a destructive midline condition only until specific laboratory or histological diagnosis are obtained and not form a basis for therapy. Once the other causes of midline destruction have beeneliminated, this disorder should be classified as a T-cell lymphoma, based on modern cytogenetic, immunologic and molecular studies.[9]

In 1982 Ishii *et al* first recognized the presence of tumor cells expressing CD3 in this lesion and termed this disease “nasal T-cell lymphoma”.[10] Further characterization of this tumor revealed angiocentric infiltration of tumor cells and the terminology of “angiocentric T-cell lymphoma” was proposed. In the REAL Classification, this type of nasal lymphoma was considered an angiocentric lymphoma, together with pulmonary lymphomatoid granulomatosis of B-cell origin, based on morphological features. However, Suzumiya *et al*. demonstrated that tumor cells of this nasal lymphoma express cytoplasmic CD3 and CD56, but not T-cell receptors, suggesting their NK-cell origin.[11] Until recently, however, it has remained obscure whether angiocentric lymphoma with the NK-cell profile is a true NK-cell neoplasm or a bonafide T-cell neoplasm with aberrant expression of NK-cell markers; hence, a few investigators have preferred the noncommittal lineage designation NK/T-cell lymphoma rather than putative NK-cell lymphoma.[12,13] On November 11-14, 1994, a workshop on NK-cell lymphomas was held in Hong Kong. At this meeting, tumor angiocentricity was not considered an absolute characteristic of nasal NK-cell lymphomas, and similarities with non-nasal NK-cell lymphomas were confirmed.

Thus, the nomenclature of “nasal and nasal-type T/NK-cell lymphoma” was employed. In the WHO classification, the extranodal origin of this lymphoma was emphasized, and the terminology “ENKL, nasal-type” was adopted.

Diagnosis of these lymphomas is dependent on a series of laboratory studies, particularly cultures, but frequently a biopsy is required to establish a diagnosis.

The interpretation of biopsy specimen, however, generally is not simple even when the pathological changes appear non-neoplastic. For example, in the setting of inflammation in the nose, paranasal sinuses and hard palate one may never be completely certain whether the biopsy specimen is representative or whether neoplastic cells have been masked by secondary inflammation and necrosis common to this anatomical site. Therefore, a diagnosis of malignant lymphoma is frequently dependent on the identification of atypical hematopoietic cells amid an intense inflammatory, necrotic and degenerating cellular milieu. Under these conditions, it is essential that the biopsy be of sufficient size and adequate technical quality in order to allow the identification of atypical cells. If atypical cells are not apparent, the lesion may be idiopathic midline destructive disease, a localized, destructive inflammatory process that is confined to the upper respiratory tract.[7] Even though angiocentric T-cell lymphoma often does not have the classic histopathological features of lymphoma microscopically, it behaves in a malignant fashion and responds to the same treatments to which lymphomas respond.[9]

### Therapy for localized extranodal NK-cell lymphoma

Radiotherapy alone has been used for the treatment of limited stage of ENKL, but the 5-year overall survival (OAS) is approximately 50%.

Recently, several groups have treated patients with irradiation of more than 45-50 Gy followed by short courses of chemotherapy, and the reported 5-year OAS of this procedure have reached 70%. However, the initial radiotherapy may miss underlying minimal lesions outside the radiation field. Therefore, a strategy of simultaneous chemoradiotherapy, as used for solid tumors, such as esophageal, laryngeal and lung cancers, has been thought to be beneficial for the treatment of ENKL. Currently, the Japanese Clinical Oncology Study Group is performing a prospective evaluation for localized nasal NK/T-cell lymphoma.

### Therapy for advanced ENKL

Most patients with advanced disease tend to be treated with chemotherapy, such as CHOP (cyclophosphamide, doxorubicin, vincristine, prednisone) or third-generation anthracycline-containing regimens, but most patients respond poorly and die within several months. Several reports demonstrated successful treatment using hematopoietic stem cell transplantation (HSCT) for these diseases. Currently,

HSCT is the only therapy expected to be curative in advanced cases. However, the results of transplant during relapse are poor, and this requires the development of more effective chemotherapeutic regimens for NK-cell neoplasms.

Recently, the NK-cell tumor study group started a phase I trial of a new combination chemotherapy named SMILE. The SMILE regimen consists of a steroid hormone, methotrexate, ifosfamide, L-asparaginase and etoposide and is a dose-finding study for methotrexate and etoposide.[2]

## CONCLUSIONS

Nonspecific nasal symptoms often predate the appearance of mucosal ulceration and tissue necrosis by one year or more. The ambiguous nature of these symptoms can result in a delay in diagnosis. Representative biopsy material and good interaction with the pathologist is important. Although not always possible, a diagnosis should be sought prior to commencing a treatment course. Previously angiocentric lymphoma would have been classified under the clinical spectrum of lethal midline granuloma, but these terms include a variety of heterogenous diseases with no attempt to delineate the underlying pathology. It needs to be understood that the term midline lethal granuloma should be a clinical term used only until specific laboratory or histological diagnosis is obtained, and not form a basis for therapy. In conclusion, clinicians should consider extranodal nasal lymphoma as a rare cause of midline destructive lesions. They should be aware of the difficulties of obtaining histological diagnosis despite apparently adequate biopsies.
